# Macrophage Phenotyping in Atherosclerosis by Proteomics

**DOI:** 10.3390/ijms24032613

**Published:** 2023-01-30

**Authors:** Sonia Eligini, Erica Gianazza, Alice Mallia, Stefania Ghilardi, Cristina Banfi

**Affiliations:** 1 Centro Cardiologico Monzino I.R.C.C.S., 20138 Milan, Italy; 2 Dipartimento di Biologia e Biotecnologie “Lazzaro Spallanzani”, Università di Pavia, 27100 Pavia, Italy

**Keywords:** atherosclerotic plaque, macrophage, proteomics

## Abstract

Macrophages are heterogeneous and plastic cells, able to adapt their phenotype and functions to changes in the microenvironment. They are involved in several homeostatic processes and also in many human diseases, including atherosclerosis, where they participate in all the stages of the disease. For these reasons, macrophages have been studied extensively using different approaches, including proteomics. Proteomics, indeed, may be a powerful tool to better understand the behavior of these cells, and a careful analysis of the proteome of different macrophage phenotypes can help to better characterize the role of these phenotypes in atherosclerosis and provide a broad view of proteins that might potentially affect the course of the disease. In this review, we discuss the different proteomic techniques that have been used to delineate the proteomic profile of macrophage phenotypes and summarize some results that can help to elucidate the roles of macrophages and develop new strategies to counteract the progression of atherosclerosis and/or promote regression.

## 1. Introduction

Cardiovascular diseases (CVDs) are the major cause of morbidity and mortality worldwide, and atherosclerosis is the main pathological condition underlying these diseases [1]. Atherosclerosis is a chronic and progressive inflammatory disease of the medium and large arteries characterized by the formation of atherosclerotic plaques. Atherosclerotic lesions originate from abnormal retention of lipids within the intima of the arterial wall with consequent stimulation of the vascular cells to produce inflammatory mediators and cytokines that attract circulating monocytes to the site of the lesion [2]. Infiltrated monocytes can differentiate into macrophages that, through scavenger receptors, internalize native and modified lipoproteins to become cholesterol-rich “foam cells” [3]. The progressive accumulation of lipids, lipoproteins, and inflammatory cells leads to the formation of a fatty streak, which subsequently evolves into an advanced lesion and atheroma [4]. The progression of the pathology is slow and, usually, atherosclerosis remains asymptomatic for several years. However, the continuous plaque growth reduces the vessel lumen to the extent of obstructing coronary blood flow, causing stable angina pectoris. This event is rarely fatal, but obstructive and nonobstructive atherosclerotic plaques may undergo erosion or rupture, and precipitate in clinical complications such as ischemia, myocardial infarction, and cardiovascular death [5,6]. 

Immune cells, especially macrophages, are critical components of atherosclerotic plaque [7]. Plaque macrophages derive mainly from the differentiation of circulating monocytes that are recruited from the circulation, and through a transmigration process involving adhesion molecules and chemotactic factors, infiltrate the arterial wall. In addition, recently, it has been shown that the resident macrophage population may be maintained also through local proliferation [8,9]. 

Macrophages participate in all the stages of the atherosclerotic lesion, from the onset to the progression and rupture. Indeed, macrophages contribute to inflammation, lipid accumulation, formation of the necrotic core, and degradation of the fibrous cap leading the plaque to rupture [10]. 

However, macrophages are a population heterogeneous and plastic, and recently, it has been shown that they can also contribute to stabilizing existing atherosclerotic plaques and even promote their regression [11].

This review discusses the different roles of macrophages in atherosclerotic plaque and examines the proteomic techniques that can help to better understand the progression of the disease and to suggest new strategies to promote plaque stabilization and/or regression. The proteomic profile provides potential mechanistic insights and candidate biomarkers for predicting diverse aspects of the pathology, such as stable versus unstable atherosclerotic plaque, suggesting their behavior, and guiding treatment. 

## 2. Macrophage Phenotypes

**Figure 1:**
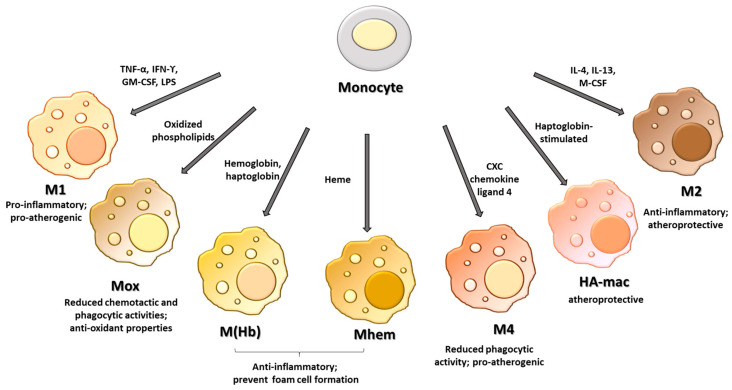
Macrophage phenotypes in atherosclerotic plaque. The different microenvironments present in atherosclerotic plaque drive the differentiation of infiltrated monocytes toward different macrophage phenotypes. TNF-α: tumor necrosis factor-α; IFN-γ, interferon-ϒ; GM-CSF, granulocyte-macrophage colony stimulating-factor; LPS, lipopolysaccharides; IL-4, interleukin-4; IL-13, interleukin-13; M-CSF, macrophage colony-stimulating factor.

In response to microenvironmental stimuli, such as growth factors, cytokines, and chemokines, macrophages differentiate into distinct phenotypes (Figure 1). Initially, macrophages were classified as classical or M1 macrophages, with proinflammatory properties, and alternative or M2 macrophages, which show anti-inflammatory characteristics. In particular, cytokines including tumor necrosis factor-alpha (TNF-α), interferon-gamma (IFNγ), granulocyte-macrophage colony-stimulating factor (GM-CSF), or bacterial products such as lipopolysaccharides (LPS) polarize macrophages toward the classical phenotype, while alternative macrophages are induced by cytokines such as interleukin (IL)-4 and IL-13 or macrophage colony-stimulating factor (M-CSF) [12]. 

M1 macrophages produce high levels of proinflammatory cytokines including IL-12, IL-23, IL-6, IL-1β, IL-8, and TNF-α, and low levels of the anti-inflammatory cytokine IL-10. Moreover, they show enhanced microbicidal activity and release high levels of reactive oxygen species and nitrogen radicals [13].

In contrast to proinflammatory M1 macrophages, the M2 phenotype shows high levels of transforming growth factor (TGF)-β and IL-10 and low levels of IL-12 and IL-23. M2 macrophages express high levels of mannose receptor (CD206) and promote wound healing through the efferocytosis process, matrix remodeling, and recruitment of fibroblasts [14,15]. M1 and M2 classification derives from in vitro observations and refers to the extreme opposite within a broad spectrum of different macrophage phenotypes. Today, this classification appears to be an oversimplification of the complex heterogeneity of macrophage phenotypes, especially in atherosclerotic plaque, where the multifaceted microenvironment contributes to inducing macrophage polarization toward other phenotypes than M1 or M2. Especially, four different subtypes of M2 phenotypes have been defined. M2a macrophages are induced by IL-4 or IL-13, they promote cell growth and tissue repair, and they are characterized by high endocytic activity and increased expression of CC chemokine ligand (CCL)17, CCL18 and CCL22. M2b macrophages are induced by immune complexes, IL-1β, and toll-like receptors and modulate the immune and inflammatory responses. Compared to other M2 macrophages, they show the ability to produce both anti- and pro-inflammatory cytokines such as IL-10, IL-6, IL-1β, and TNF-α [16]. The M2c phenotype or inactivated macrophages are induced by TGF-β, IL-10, and glucocorticoids. They secrete high levels of CCL16 and CCL18, and show high efferocytotic capability [17]. Finally, M2d macrophages, obtained after stimulation with toll-like receptor and adenosine A_2A_ receptor agonists, produce high levels of vascular endothelial growth factor and low levels of IL-12 and TNF-α. Moreover, in contrast to other M2 phenotypes, this subgroup does not express high levels of the CD206 receptor [18,19].

In addition to these phenotypes, the complex microenvironment of the atherosclerotic lesion induces the differentiation of resident macrophages toward other phenotypes including macrophages that respond to oxidized phospholipids (Mox), hemoglobin-related phenotypes such as hemoglobin-stimulated (M(Hb)), haptoglobin-stimulated (HA-mac) and heme-stimulated (Mhem) macrophages, and M4 macrophages. Macrophages residing in the atherosclerotic plaque are continuously exposed to lipids and their oxidized products that accumulate in the lesion. In this context, it has been reported that in response to oxidized phospholipids, macrophages are polarized toward a phenotype called Mox. Mox polarization involves the activation of the transcription factor nuclear erythroid-2-related factor, and compared to M1 and M2 macrophages, Mox phenotypes show different morphology and functions and are characterized by reduced chemotactic and phagocytic activities. In addition, Mox macrophages express several antioxidant enzymes such as heme oxygenase-1, thioredoxin reductase 1, sulfiredoxin 1, and glutathione reductase-1 [20]. In a previous paper, we demonstrated that the treatment with oxidized phospholipids of macrophages obtained in vitro from the differentiation of human monocytes inhibits the expression of cyclooxygenase-2 [21], an enzyme that has been detected in atherosclerotic plaque, mainly in macrophages [22]. In murine atherosclerotic lesions, Mox macrophages account for about 30% of the total macrophages; however, the presence of this phenotype in human atherosclerotic plaque has not yet been reported. 

In the hemorrhagic areas of human atherosclerotic plaque, different sub-populations of macrophages related to the presence of hemoglobin and erythrocytes have been identified. M(Hb) macrophages express high levels of CD206 and CD163, the scavenger receptor for the hemoglobin/haptoglobin complex, which is required for efficient hemoglobin clearance after intraplaque hemorrhage [23]. After ingestion of erythrocytes, the released heme group can promote macrophage polarization toward a Mhem phenotype, with consequent activation of the activating transcription factor 1. This activation leads to the expression of heme oxygenase-1 (HO-1), liver X receptor (LXR)-α, and ATP-binding cassette transporter ABCA1, which show atheroprotective functions and prevent foam cell formation [24,25]. HA-mac macrophages show high levels of CD163 and HO-1, but low levels of human leukocyte antigen-DR [26]. 

Macrophages polarized by CXC chemokine ligand 4 generate M4 macrophages characterized by the expression of metalloproteinase (MMP) 7 and the calcium-binding S100-A8, the lack of expression of CD163, and reduced phagocytic activity [27,28].

## 3. Role of Macrophage Phenotypes in the Atherosclerotic Plaque

The progression of atherosclerotic plaque, as well as its activity, is associated with an increase in the total number of macrophages resident in the plaque [29]. In particular, both M1 and M2 macrophages increase with plaque growth, and the number of total intraplaque macrophages is greater in symptomatic than asymptomatic lesions [29,30]. As each macrophage phenotype exhibits different properties and explicates different functions, the prevalence of a phenotype may profoundly affect the progression, stabilization, or regression of the plaque. It has been shown that macrophages located in the plaque shoulder, a site prone to rupture, display mainly a proinflammatory phenotype and express M1 markers, while macrophages positioned in the fibrous cap express both M1 and M2 markers [29]. Thus, while M1 macrophages sited in the fibrous cap contribute to destabilizing the plaque through the production of MMP, M2 macrophages can partially counteract this damaging effect through the release of pro-fibrotic factors such as fibronectin, insulin-like growth factor, and TGF-β, and contribute to stabilizing the plaque [31]. In accordance, both symptomatic and asymptomatic stable plaques show a prevalence of M2 macrophages. 

In contrast, M1 macrophages are more abundant in vulnerable plaques [30].

In a study by de Gaetano et al., human carotid atherosclerotic plaques isolated from symptomatic and asymptomatic patients were analyzed by real-time PCR and Western blot analysis. By analyzing the cellular content and distribution of M1 and M2 macrophage phenotypes, they showed that in asymptomatic plaques, compared to symptomatic ones, the expression of CD68 was decreased 3-fold, the expression of ABCA1 was decreased 2.7-fold, and the expression of the M2 marker CD206 was increased 2-fold. Moreover, they showed that M2 macrophages were relatively abundant in asymptomatic plaques (42 ± 5% of the total macrophage population) and were only 23 ± 3% in symptomatic plaques [32]. On this basis, it is possible to hypothesize that the balance between M1 and M2 macrophages, as well as their distribution into the plaque, can profoundly affect the fate of the lesion. 

In addition, exposure to the different microenvironments of atheroma induces macrophages toward other phenotypes. In response to an intraplaque hemorrhage, macrophages are polarized toward M(Hb) and Mhem phenotypes. These phenotypes are involved in the clearance of hemoglobin with the subsequent production of anti-inflammatory cytokines, such as IL-10 [23,33]. Moreover, M(Hb) and Mhem phenotypes show increased expression of the nuclear receptor LXR-α and ATP-binding cassette transporter, which increase the cholesterol efflux and prevent foam cell formation [34]. In contrast, M4 macrophages show a pro-atherogenic profile and participate in the complications of atherosclerosis such as thrombosis and acute coronary syndrome as they produce the proinflammatory cytokines IL-6 and TNF-α, and MMP7 and MMP12 that degrade the extracellular matrix proteins and contribute to the destabilization of atherosclerotic plaque [28,35].

On this basis, thorough knowledge of the different phenotypes and their abundance in the plaque may be important for predicting the clinical course and preventing fatal events. As proteins are the main effectors of major biological processes, the proteomic profile may be a powerful tool to identify the complex molecular pathways in multifactorial diseases including atherosclerosis. 

Several public databases related to proteomic studies performed on human and/or animal macrophages are available in ProteomeXChange (http://proteomecentral.proteomexchange.org/cgi/GetDataset (accessed on 20 December 2022)), and can provide useful information for further re-analyses. Our lab also contributed to disseminating proteomic results by publishing two manuscripts containing the list of identified proteins in human macrophages spontaneously differentiated in vitro into two main morphotypes [36,37].

## 4. Methods to Study Plaque Macrophage Phenotypes 

**Figure 2:**
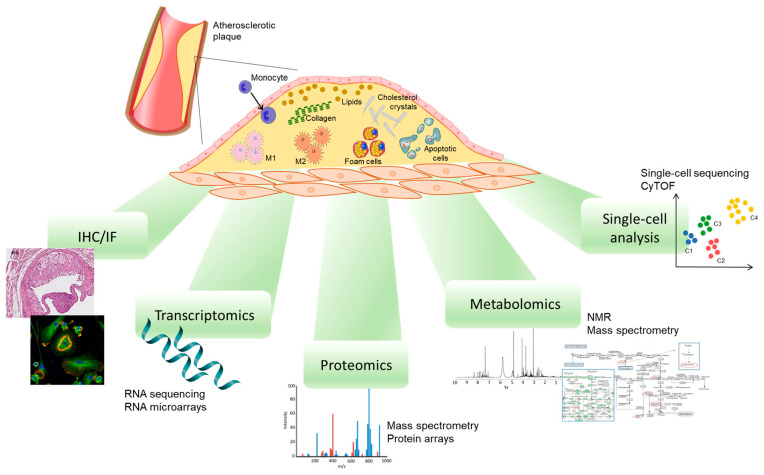
Different techniques to study macrophage phenotypes. IHC/IF, immunohistochemistry/immunofluorescence; NMR, nuclear magnetic resonance; CyTOF, cytometry by time of flight.

Although it is widely known that a variety of macrophage phenotypes coexist simultaneously in the plaque, their specific characteristics, as well as their functions, are poorly understood. Thus, in recent decades, several approaches have been used to describe the phenotype, localization, and distribution of macrophages resident in the atherosclerotic plaque, including immunohistochemistry or immunofluorescent studies, the transcriptome, and proteomics studies [29,32,38] (Figure 2).

Even if the identification of the different phenotypes of macrophages by immunohistochemistry provided important information regarding the heterogeneity of the cell populations resident in plaque, this methodology shows some limitations. For example, it is known that smooth muscle cells in atherosclerotic plaque can express macrophage markers, making the discrimination of the real macrophages difficult and leading to misidentification [39]. Moreover, several constituents of atherosclerotic plaque can generate autofluorescence, affecting the specific signal and causing false positive signals. In particular, macrophages can display autofluorescence, which varies according to the differentiation state of the cell [40]. Similarly, oxidized lipids such as ceroids, which accumulate in the necrotic core, show an intensive autofluorescence [41,42]. Thus, ruptured plaques and plaques at high risk of rupture can show near-infrared autofluorescence derived from intraplaque hemorrhage and heme degradation [43].

The different phenotypes of macrophages have been well characterized through the use of several methods based on gene expression analysis [44,45,46,47]. Transcriptomics studies have provided a wealth of information about the markers of polarization and have shown that each macrophage phenotype is associated with a specific gene profile [48]. In addition, single-cell RNA sequencing has further improved the knowledge about the heterogeneity of the macrophage population [49,50,51]. However, it has been shown that macrophages are more easily damaged than other cells during the enzymatic and mechanical dissociation processes required for single-cell RNA sequencing studies, resulting in an underestimation of macrophages [52]. Moreover, as not all the transcripts are detected in all cells, some cells may be false negatives [49]. Finally, it is necessary to emphasize that gene expression does not always correlate with the levels of the protein and/or with its activity. 

Therefore, the study of protein expression is expected to provide relevant information about macrophage heterogenicity. Several proteomic technologies are available and constantly evolving, and the recent advances in mass spectrometry (MS) helped to gain increasingly comprehensive insights into the proteome profiling of atherosclerotic plaque macrophages. Sample preparation has an important role in proteomic characterization and optimization, and standardization of the analytical procedures is required based on the proteomic complexity, the available quantity of the sample, and the aim of the study [53]. The proteome profiling involves appropriate isolation and purification techniques before the MS analysis. The classical and widely used approach includes two-dimensional gel electrophoresis (2-DE) coupled with MS and its variants such as difference gel electrophoresis, which provides greater sensitivity and reproducibility in the proteome characterization [54]. However, this technique is time-consuming and poorly consistent as well as having great limitations, such as the presence sometimes of multiple proteins under the same single gel spot and the inability to properly detect proteins with high molecular weight, hydrophobic proteins, and extremely acid and basic proteins [55]. Gel-free MS-based technology is more robust and reliable and led to an improvement in proteome analysis thanks to the development of high-throughput separation and analytical strategies, among which those mainly used in proteomics are matrix-assisted laser desorption/ionization time-of-flight MS (MALDI-TOF MS) and liquid chromatography coupled with tandem mass spectrometry (LC-MS/MS). MS-based proteomics techniques are often used for large-scale protein identification, and, in particular, shotgun proteomics is increasingly applied not only for discovery studies but also for quantification [56]. 

Shotgun proteomics is the most widely used MS-based method for unbiased protein quantitation from biological samples [57] and consists of a multidimensional separation of peptides in complex biological samples, obtained from the sample treatment with specific proteases, and their analysis using MS/MS to determine a global protein profile of the sample.

To collect quantitative data, MS-based proteomic studies can be performed based on stable isotope labeling (e.g., stable isotope labeling with amino acids in cell culture (SILAC), the isotope-coded affinity tag (ICAT), and isobaric tags for relative and absolute quantitation (iTRAQ)) [58] or label-free techniques (e.g., precursor signal intensity and spectral counting) [59] for relative and absolute quantification. Label-free analysis requires a very small sample amount, minimal sample manipulation, and lower costs. Moreover, label-free protein quantification is ideal for the large-scale characterization of proteomes and biomarker discovery studies. 

The high-throughput ability of the multiple reaction monitoring (MRM) technique [60] and protein arrays [61] allows also the validation of particular proteins of interest, understanding better the roles of individual proteins and their abundances, as well as applying this approach to several proteomics areas such as biomarker discovery and pharmacological studies for CVDs. 

A challenge in studying plaque-associated macrophages is their effective isolation within the complexity of lesions. Pathological samples often contain a mixture of different cells [54]. Indeed, the study of specific regions or cell types from atheroma plaques provides subproteomes that yield more detailed information and differences in protein expression, which are not possible to distinguish in a global analysis of the whole plaque tissue [62]. Several isolation techniques have been developed, such as microdissection, explants cultures, and cell sorting, but they are not always applied to proteomic studies, due to the low amount of cell/tissue obtained. 

The applications and the advantages/disadvantages of cell isolation by laser microdissection and cell sorting are depicted in Table 1.

In the future, the attention will be focused on the development or improvement of analytical tools that are increasingly sensitive and reliable in protein isolation and detection from particular cell subproteomes [62], such as the complex heterogeneity of macrophage phenotypes.

## 5. The Proteomic Analysis of Atherosclerotic Plaque Highlights the Contribution of Macrophages

The atherosclerotic plaque is a complex structure composed of several cell types with different phenotypes and in which the cell–cell interaction profoundly affects the behavior of the plaque. The proteomic analysis of the plaque can provide a broad view of the proteins involved in atherosclerosis. The abundant presence of proteins produced by macrophages in atherosclerotic plaque further confirmed the important role played by these cells. Below, we focus on proteins that are produced and/or can modulate the macrophage roles in the plaque. Among the proteins differentially expressed in the atherosclerotic and non-atherosclerotic samples obtained from human carotid endarterectomy and analyzed by Western blot using 823 monoclonal antibodies, a marked downregulation of a proapoptotic protein (apoptosis-linked gene 2) was evidenced, suggesting a mechanism by which macrophages resist apoptotic signaling and survive in the atherosclerotic plaque [63]. 

Using protein microarray technology, the protein profile of human endarterectomy of stable and unstable carotid plaques has been compared. The results have evidenced the deregulation of proteins associated with unstable plaque regions, and in particular, the authors identified overexpression of caspase-9 and TNF receptor-activating factor-4 in macrophage-rich regions from unstable hemorrhagic and ulcerated plaques, suggesting increased macrophage apoptosis [64]. While, in the early lesion, macrophage apoptosis reduces the plaque progression, in the advanced lesion, it promotes the growth of the necrotic core, rendering plaque prone to rupture [65]. In addition, a marked increase in Grb2-like adaptor protein detected in macrophages of unstable plaque suggests an increase in the differentiation process of monocytes into macrophages during unstable plaque development [64].

The analysis of 35 human coronary atherosclerotic plaques using direct tissue proteomics (DTP) by LC-MS/MS has allowed the identification of a total of 806 proteins providing the first large-scale proteomics map of human coronary atherosclerotic plaques. Among them, it has been shown that annexin I was expressed in macrophages resident in the tunica intima that showed a foam cell phenotype [66]. Moreover, in this study, the authors demonstrated that the DTP method was compatible with laser capture microdissection, and the absolute quantitation of specific low-abundance cytokines and growth factors in the human coronary arteries was possible by the MRM technique.

Recently, transcriptomic and proteomic profiles have been defined in stable and unstable human carotid atherosclerotic plaques obtained by carotid endarterectomy. Proteome analysis was performed by LC-MS/MS and 3082 proteins were identified, among which 293 were differentially expressed in stable and unstable plaques [67]. In particular, increased levels of CD5L, a glycoprotein mainly expressed in macrophages that shows antiapoptotic properties, supports foam cell formation, and polarizes macrophages toward the M2 phenotype, were detected in unstable plaques. Moreover, unstable plaques showed upregulation of S100A12, a proinflammatory protein able to bind the receptor for advanced glycation end products, and toll-like receptor 4, activating the downstream proinflammatory signaling [67]. In addition, S100A12 contributes to the progression of atherosclerosis by the recruitment of monocytes and mast cells [68].

A proteomic study on homogenates of coronary atherosclerotic plaques at different stages of development was performed by 2-DE and MALDI-TOF MS. The authors showed increased levels of the cytoskeletal proteins microfibril-associated glycoprotein 4 and vimentin in the stable atherosclerotic plaque at the stage of lipidosis and fibrosis [69]. In particular, vimentin is abundantly expressed in macrophages during atherogenesis and in foam cells. Vimentin deficiency in macrophages is associated with an increase in CD36 expression and increased uptake of oxidized low-density lipoprotein (oxLDL) [70]. The progression of stable atherosclerotic plaque to the stage of fibrosis and calcification induces a reduction in the concentration of these cytoskeletal proteins, and a significant increase in the mimecan and fibrinogen amounts [69].

Recently, an unbiased proteomic study was performed on the aortas of a transgenic mouse model in which the human scavenger receptor promoter drives macrophage-specific overexpression of the mouse urokinase-type plasminogen activator gene [71]. In parallel, the authors also performed shotgun proteomics by LC-MS/MS analysis on extracts of ruptured and stable regions of freshly harvested human carotid plaques. The analysis identified several proteins and biological pathways associated with the rupture, such as loss of plaque basement-membrane proteins, extracellular proteolysis, inflammation, and a decrease in cell-matrix adhesion, which were confirmed in the extracts of ruptured human carotid plaques [71]. 

## 6. The Analysis of the Atherosclerotic Plaque Secretome Reveals the Contribution of Macrophages

Another application of proteomics is the study of proteins and microparticles released from the atherosclerotic plaque in the bloodstream, which could be important in cell signaling, communication, and regulation of several pathophysiological processes. The secretome is highly dynamic because it reflects the state of the cells in real-time and at different stages of a pathological condition, allowing the discovery of potential protein biomarkers [72,73]. The analytical procedure involves several steps, including the cell culturing and conditioning, the conditioned medium collection and concentration/fractionation by different protein separation methods, and, finally, the identification of secreted proteins by MS. This paragraph focuses on the contribution of macrophages to the secretome of atherosclerotic plaque with mediators that contribute to the formation of a vulnerable plaque or, in contrast, to the anti-inflammatory effects. In several studies, atherosclerosis was studied by evaluating protein secretion from foam cells, suggesting that cytokines and other factors can regulate disease progression [74,75,76].

Duran and colleagues, who were the pioneers in the analysis of the plaque secretome, demonstrated that atherosclerotic plaques are not immutable and can be used as a tool to find novel disease markers [77,78]. Secretomes from human atherosclerotic plaques incubated in the presence or absence of atorvastatin were compared with those of adjacent fibrous areas used as controls by 2-DE, to identify released proteins that potentially correlate with atheroma plaque formation and rupture [77]. Several proteins were identified by MALDI-TOF MS, some with higher or lower levels in the atheroma plaque supernatants compared to controls [77]. Among these significant proteins, for example, cathepsin D, which was highly expressed in macrophage-rich areas of advanced carotid atherosclerotic plaques, reverted to control values after administration of atorvastatin. Of interest, cathepsin D has a key role in plaque vulnerability and rupture, because it is involved in the degradation of the extracellular matrix of atherosclerotic plaques and the apoptosis of macrophage-derived foam cells [79].

The evaluation of human secretomes from a cultured unstable carotid atherosclerotic plaque and non-atherosclerotic mammary artery showed significantly higher levels of the adipokine visfatin in the unstable plaques [80]. Indeed, visfatin is a potential inflammatory mediator, localized to foam cell macrophages within unstable atherosclerotic lesions, which promotes cholesterol accumulation, atherosclerosis development, and plaque vulnerability [81,82]. Moreover, it has been shown that the combined stimulation by oxLDL and TNF-α in symptomatic atherosclerotic plaques markedly increased visfatin expression [81]. 

In another study by the same group of authors, the protein secretion profiles of the carotid atherosclerotic plaque and non-atherosclerotic mammary artery were compared using an untargeted proteomic approach that involved tandem immunoaffinity depletion, iTRAQ labeling, and nanoLC-MS/MS [83]. About 160 proteins were quantified, among which neutrophil defensin 1, apolipoprotein E, and clusterin showed increased levels in the carotid secretome. Neutrophil defensins can induce leukocyte transendothelial migration and increase foam cell formation [84], while clusterin with cytoprotective and anti-inflammatory properties has an upregulated expression in the human aorta with the progression of atherosclerosis [85]. Instead, apolipoprotein E may have a protective role in macrophages by promoting cholesterol efflux from cells of the arterial wall [86].

## 7. Proteomic Analysis of In Vitro-Cultured Macrophages

All the cell types resident in the atherosclerotic plaque participate in different ways in the atherosclerotic process, and studying the individual constituents of plaque may provide important information about the contribution of each cellular element. The study of an individual constituent may be performed using in vitro cell models or by the isolation of a specific cell type.

Some studies have characterized, through proteomic analysis, the different macrophage phenotypes obtained in vitro from human or animal models [87,88,89]. The use of cultured cells shows the advantage to select a cell type that is cultured in a specific microenvironment using different stimulant agents. By a mass spectrometry analysis, a global view of the proteome of classically or alternatively activated murine macrophages generated in vitro after exposure to M-CSF or GM-CSF was provided. The authors identified 106 proteins that were enriched in the membrane of the different phenotypes and delineated a unique signature that distinguishes the two types of macrophages [90]. Further, different studies have investigated the role of oxLDL on protein expression in macrophages using human cell lines such as THP-1 or U937 [61,91,92]. The protein array analysis of oxLDL-stimulated THP-1 cells showed increased expression of the scavenger receptor CD36, the inflammatory protein cyclooxygenase-2, cyclin-dependent kinase 1, transcription factor II-I, NEMO-like kinase, and Elf-5 that might play a role in cell proliferation and cell adhesion [61]. 

By using 2-DE were detected more than 2500 spots in THP-1 monocytes treated with oxLDL or LDL compared to non-treated THP-1. About 150 spots were differentially expressed, and 93 of them were identified by MALDI-TOF analyses. Among these proteins, a marked increase was detected for vimentin, suggesting a role in the uptake and retention of oxLDL. Moreover, increased levels were also evidenced for annexin I, which is overexpressed both in phorbol 12-myristate 13-acetate-differentiated THP-1 and human macrophages obtained from the differentiation of monocytes [92]. 

U937 cells incubated with oxLDL were used as a model of foam cells for proteomic studies using 2-DE and tandem mass spectrometry. The interested protein spots were excised from the gel, in-gel trypsin digested, and identified by LC-MS/MS. The uptakes of oxLDL are associated with the upregulation of proteins involved in carbohydrate and lipid metabolism. In particular, the increased levels of enzymes such as aldolase, glyceraldehyde-3-phosphate dehydrogenase, and peroxiredoxin-1 confirmed the alteration of lipid metabolism and the oxidative stress detected in foam cells [91]. 

Human macrophages obtained from the differentiation of circulating monocytes were also used to define the macrophage proteome in different states of polarization. Using a label-free quantification approach by nanoLC-MS/MS in gel-fractionated and trypsin/LysC-digested samples, Court et al. identified and quantified 5102 proteins [93]. Each polarization state showed specific proteins. Macrophages obtained by the differentiation of human monocytes exposed to M-CSF, and defined as unstimulated because no further stimulus was added, showed an upregulation of chitotriosidase-1, a phagocyte-specific chitinase with an important role in the immune response whose levels markedly increase during the differentiation of monocytes to macrophages [94], and cystatin-C, an endogenous cysteine protease inhibitor with anti-atherogenic properties [95]. In contrast, unstimulated macrophages, compared with other polarized macrophages, showed lower levels of arachidonate 15-lipoxygenase B [93], an enzyme that is involved in intracellular lipid accumulation and foam cell formation [96]. Macrophages polarized with IFNγ + LPS showed an upregulation of the M1 marker CD38, while an alternative activation induced by IL-4 + IL-13 showed a downregulation of proteins involved in the toll-like receptor 4-mediated response to LPS and upregulation of several major histocompatibility complex class II proteins with protective functions in atherosclerosis [93,97].

Recently, using label-free quantitative proteomics, it has been demonstrated that induced pluripotent stem cells (iPSCs)-derived macrophages polarized toward M1 or M2 phenotypes showed distinct profiles and different functions. In addition to confirming that M1 macrophages were mainly involved in pathogen defense, and M2 was more efficient at migrating to the site of wound healing, the authors identified novel cell surface proteins and changes in intracellular signaling molecules, expression of transcriptional factors, and secretion of chemokines and cytokines. Altogether, this study provides new knowledge on how polarization differentially modulates the macrophage functions [98]. Although all these proteomic studies provided the basis for more focused studies, and allowed for generating and testing several hypotheses, monocyte/macrophage cell lines as well as macrophages obtained from differentiation of circulating monocytes may not be representative of macrophages resident in the plaque, as they are in a static situation and do not reflect the dynamism of the in vivo conditions. Therefore, macrophages obtained directly from atheroma surely represent the ideal source for studying these cells, but, unfortunately, these samples are heterogeneous and contain other cell types.

## 8. Single-Cell Proteomics of Macrophages

The discovery of single-cell technologies has allowed a detailed analysis of the different cell types present in atherosclerotic plaque and has provided a wide view of the cellular alterations associated with clinical complications. In particular single-cell studies have revealed the complex heterogeneity, the high specialization, and the plasticity of the resident cells in the plaque. In this scenario, the combination of single-cell RNA sequencing and protein analysis has allowed us to recognize specific subsets of macrophages and define their molecular and functional profiles [50,99,100]. 

In our experience, macrophages obtained in vitro following a spontaneous differentiation of monocytes are heterogenous in functions and morphology, with two distinguishable dominant morphotypes: round- and spindle-shaped cells. Taking advantage of laser microdissection, our group isolated and delineated the proteomic profile of these two subsets, and using LC-MS^E^, we identified 28 proteins that were more abundant in spindle cells, and 28 that were more abundant in the round macrophages. Spindle macrophages showed a prevalence of proteins involved in membrane traffic regulation, while round macrophages showed a prevalence of proteins involved in efferocytosis, lipid handling, and protection against stress conditions [36,37]. These results, thus, reinforced the functional heterogeneity of the two different morphotypes.

Mass cytometry, or cytometry by time-of-flight, is a powerful single-cell proteomic analysis technique that provides simultaneous quantitative detection of 40–50 cellular parameters at the single-cell and is suitable for defining the biological profile of circulating cells, as well as tissue-resident cells [101]. Indeed, mass cytometry is a variation in flow cytometry coupled to TOF mass spectrometry, which uses antibodies conjugated with specific metal isotopes instead of fluorophores for labeling cellular proteins [102], thus overcoming the limited multiplexing capability of fluorescence-activated cell sorting.

Using single-cell RNA-sequencing and mass cytometry, Winkels et al. identified in mouse atherosclerotic aorta several novel leukocyte subpopulations, including two potential macrophage subsets such as CD11b^+^HLA-DR^med^ and CD11b^+^CD36^+^. In addition, they showed that the heterogeneity of the leukocyte populations in human carotid plaque after endarterectomy detected by mass cytometry analysis was comparable to that performed in mouse aortic leukocytes [103].

Recently, using mass cytometry and transcriptomic analyses, Fernandez et al. defined the profile of innate and adaptative immune cells in the plaque. The comparison between symptomatic (patients with recent stroke or transient ischemic attack) and asymptomatic patients with carotid atherosclerosis by mass cytometry showed 15 plaque-tissue-specific MetaClusters, including two macrophage subsets with classically activated M1 macrophages and alternatively activated M2 macrophages characterized by high levels of CD206 and CD163 receptors. The single-cell transcriptional analysis of plaque macrophages also identified the presence of five different clusters. In particular, macrophages obtained from plaques of asymptomatic patients showed enrichment in genes involved in the regulation of lipid metabolism and foam cell formation. In contrast, macrophages obtained from plaques of patients with recent clinical events displayed fewer pro-inflammatory genes, genes associated with plaque instability, and genes involved in the healing processes. Overall, macrophages of asymptomatic patients showed a more activated and proinflammatory profile with increased foam cell properties compared to macrophages of symptomatic patients. This study has provided the first immune atlas of human atherosclerosis and identified specific features of innate and adaptive immune cells in atherosclerotic plaque [50]. 

## 9. Macrophage Proteins as Therapeutic Targets

The high plasticity of macrophages makes these cells an excellent target for the treatment of atherosclerosis or all those inflammatory conditions in which the prevalence of a macrophage phenotype may contribute to amplifying the pathology or delay the progression and/or promote the regression. Therefore, specific therapies able to modulate macrophage phenotypes are increasingly considered a powerful tool for counteracting plaque formation and its destabilization. 

Proteomic studies have the potential to identify disease-related changes in the protein content, identify potential therapeutic targets, and follow the response to pharmacological treatments.

Several drugs clinically used can affect macrophage phenotypes: metformin, a hypoglycemic drug, polarizes macrophages toward the Mhem phenotype by the activation of AMPK signaling, its transcription factor 1 (ATF1), heme oxygenase (HO-1), and the LXRβ pathway [104]; pioglitazone reduces lipid content in atherosclerotic plaque with a concomitant increase in M2 and decrease in M1 phenotypes [105]; rivaroxaban, an oral anticoagulant drug, in addition to its antithrombotic activity, reduces the progression of atherosclerosis in apoE-deficient mice, at least in part, through the inhibition of pro-inflammatory activation of macrophages [106]; sitagliptin, an anti-diabetic drug used to treat the type 2 diabetes, induces the shift in macrophages toward a M2 phenotype via the activation of the SDF-1 (stromal-cell derived factor 1)/CXCR4 (CXC chemokine receptor type 4) signaling pathway [107].

Proteins expressed on the surface of macrophages can be used to develop targeted drug delivery systems. CD163 might represent a promising target as it is selectively expressed by monocytic lineage, endocytoses the ligand within a few minutes, and recycles the receptor on the cell surface. In addition, a rapid internalization of CD163 binding antibodies has been shown [108,109,110]. Glucocorticoids may be used to polarize macrophages toward an anti-inflammatory phenotype [111]. In this respect, it has been demonstrated that anti-CD163-dexamethasone conjugate reduced the inflammation in the hepatic acute phase response in mice after LPS treatment [112] and improved the liver inflammation in fructose-induced non-alcoholic steatohepatitis [113], highlighting in vivo the anti-inflammatory activity of the conjugate. 

The mannose receptor CD206 was also considered for targeted delivery to macrophages. Indeed, He et al. demonstrated that mannose-functionalized dendrimeric nanoparticles can be used for the specific delivery of LXR ligands to macrophages. Moreover, the uptake of LXR ligands by macrophages present in the plaque reduced the plaque size and the necrosis [114].

Nanoparticles loaded with the leukemia inhibitory factor, an anti-inflammatory cytokine that inhibits the inflammatory signaling in macrophages, have been conjugated with CD11b antibody to deliver anti-inflammatory cytokines to macrophages and promote the switch from an inflammatory to an anti-inflammatory macrophage phenotype [115].

CD64 might be another attractive receptor as a target of a specific macrophage phenotype, as it is upregulated in activated macrophages at sites of chronic inflammation. In particular, it has been shown that the depletion of human M1 macrophages using CD64-targeted immunotoxins induced changes in microenvironments favoring the polarization of macrophages toward the M2 phenotype that promotes the resolution of inflammation [116]. These observations suggest that this receptor might represent an interesting tool to remove M1 macrophages, reduce the inflammatory response, and favor the resolution of the inflammatory process [117]. 

A summary of macrophage proteins modulated by drugs is shown in Table 2.

## 10. Conclusions and Future Perspectives

Macrophages play a crucial role in the onset, progression, and activity of atherosclerotic plaque. They are a complex heterogenous population with several phenotypes characterized by different and also opposing functions; thus, the possibility to define a global profile of each phenotype represents an attractive target to develop therapies directed to reduce the progression of the disease and promote its regression. Proteomics represents a powerful tool involving different high-throughput technologies in continual development, which can help in the understanding of the complexity of cells present in atherosclerotic plaque and their behavior. The proteome is a rich source of potential biomarkers that could be useful to characterize the progression of atherosclerosis and define diagnostic and therapeutic targets for plaque stabilization and/or regression [118]. The recent advances in MS techniques helped to provide more comprehensive information for developing the proteome profiling of atherosclerotic plaque macrophages. The proteomic studies directed to characterize the role of macrophage phenotypes in atherosclerosis and discussed in this review are listed in Table 3. 

In addition, the therapeutic control of macrophage metabolism can be another useful tool to affect the macrophage inflammatory state as well as to stimulate or prevent protective and pathogenic macrophage functions. Indeed, the study of metabolic changes in macrophages on atherosclerotic plaque progression and stability is an area of great interest [119], and learning mechanisms that regulate the metabolic adaptation of M1 and M2 macrophages can provide new therapeutic targets not only in atherosclerosis but also in chronic inflammatory diseases.

In recent years, single-cell technology has become an increasingly important approach to defining a detailed signature for macrophages and other immune cell subsets present in atherosclerotic plaque. This technology has greatly expanded the knowledge concerning the complex mechanisms underlying the atherosclerotic process, providing an accurate picture of the biology and heterogeneity that is present in the atheroma.

In conclusion, it is expected that an integrated “multi-omics” approach that involves transcriptomic, proteomic, and metabolomic, also associated with single-cell technologies, can significantly increase the knowledge about the role of macrophages in atherosclerosis, and help to develop targeted therapies toward a specific phenotype. 

## Figures and Tables

**Table 1:** Applications and advantages/disadvantages of cell isolation by laser microdissection and cell sorting.

Method	DNA/RNA Analysis	Proteins Analysis	Wide Range of Source Material	Labor Time	Cost	Notes
*Laser* *microdissection*	V	V	V	High	High	Long-term sample storage is not recommended. Visualization of the specimen is hampered by the absence of a coverslip. Careful handling of samples is necessary and adequate sample processing is essential for reproducible results.
*Cell sorting*	V	V	V	Medium	High	Measures single cells keeping the cell alive. Allows the simultaneous analysis of multiple parameters. Requires highly trained specialists. Tissue structure is lost. Little information on intracellular distribution.

**Table 2:** Summary of macrophage proteins modulated by drugs.

Drug	Proteins Target	Signaling	Metabolic Pathway	Reference
Metformin	HO-1	AMPK/ATF	Oxidative stress, M2 phenotype	Seneviratne A. et al., 2021
Pioglitazone	MCP1/CCR2	PPARγ	Inflammation, M2 phenotype	Tokutome M. et al., 2019
Rivaroxaban	MMP9, TNFα	FXa/PARs	Inflammation	Hara T. et al., 2015
Sitagliptin	CXCR4	SDF-1	M2 phenotype	Brenner C. et al., 2015
Anti-CD163-dexamethasone	α-2-macroglobulin	CD163	Inflammation	Thomsen K.L. et al., 2016
Anti-CD163-dexamethasone	Il-1β, TNF-α	CD163	Inflammation	Svendsen P. et al., 2017
Mannose functionalized dendrimeric nanoparticles	ABCA1, ABCG1	CD206/LXR	Lipid metabolism	He H. et al., 2018

*Abbreviations*: HO-1, heme oxygenase-1; AMPK, 5’ adenosine monophosphate-activated protein kinase; ATF1, activating transcription factor 1; MCP1, monocyte chemoattractant protein-1; CCR2, C-C chemokine receptor type 2; PPARγ, peroxisome proliferator-activated receptor-gamma; MMP-9, matrix metallopeptidase-9; TNFα, tumor necrosis factor-alpha; FXa, activated factor X; PARs, protease-activated receptors; CXCR4, C-X-C chemokine receptor type 4; SDF-1, stromal-cell-derived factor 1; IL-1β, interleukin-1; TNFα, tumor necrosis factor-alpha; ABCA1, ATP-binding cassette transporter A1; ABCG1, ATP-binding cassette transporter G1.

**Table 3:** Summary of proteomic studies performed on human macrophages.

Sample	Species	Technique	Number of Proteins Identified	Most Relevant Proteins Identified	Pathway	Reference
Carotid plaque	Human	Western blot	823	ALG-2, TSP-2, Mn-SOD, ApoE, ApoB100, PTP1C, GSK-3β	Atherosclerosis	Martinet W. et al., 2003
Carotid plaque	Human	Microarray	512	Caspase-9; clived Gads; GIT1; HIF-1α; JAM-1; JNK; L-caldesmon; c-src; TNF-α; TOPO-II-α; TRAF4	Plaque instability	Slevin M. et al., 2006
Carotid plaque	Human	LC-MS/MS	3082	CD5; S100A12; CKB; CEMIP; endophilin-B1	Plaque instability	Bao M.H. et al., 2021
Carotid plaque	Human	LC-MS/MS	1161	LAMC1; LAMA5; LAMB2; HSPG2; NID1; AGRN; NID2; COL18A1; ELN; FBLN5; LTBP4; MFAP4; BCAM	Plaque instability	Vaisar T. et al., 2020
Carotid plaque	Human	2-DE/MALDI-TOF MS	620	Cathepsin D; LRG; transferrin; apoA-I; fibrinogen; α-1-antitrypsin; protein HC; SAP; HSP27; enolase 1	Atherosclerosis	Duran M.C. et al., 2007
Carotid plaque	Human	ELISA	6	visfatin, adiponectin, IL-6, lipocalin-2, resistin, TNFR2	Plaque instability	Auguet T. et al., 2016
Carotid plaque	Human	iTRAQ labeling/nanoLC-MS/MS	162	Defensin 1; apoE; clusterin; ZAG; ecSOD; Prdx2; CA1; Hsp70	Atherosclerosis	Aragones G. et al., 2016
Coronary plaque	Human	LC-MS/MS	806	SDF1-α; TGF-β; PEDF; MFG-E8; annexin I	Atherosclerosis	Bagnato C. et al., 2007
Coronary plaque	Human	2-DE/MALDI-TOF MS	n.a.	Vimentin, tropomyosin β-chain; actin; keratin; tubulinβ-chain; MAGP4; SAP; annexin5	Atherosclerosis	Stakhneva E.M. et al., 2019
THP-1 cells	Human	Antibody Microarray	384	LAR(PTP); AKAP 149; rabaptin-5; RanBP1; NM23-H1; SIPA; CDK-1; cell division cycle 27; CLIP-115; CLIP-115; proliferation antigen Ki-67; Rnase HI; TFII-I, Kalinin B1; CD36; COX-2; p47phox; caspase-1; caspase-6; caspase-8; TRADD	Atherosclerosis	Tuomisto T.T. et al., 2005
THP-1 cells	Human	2-DE/MALDI-TOF MS	2500	vimentin; MGA6; PP2A; β-1,3-galactosyltransferase; annexin I; ferritin; TFPI 2; HFR; rho GDI-α; rho GDI-β; piridoxal kinase	Atherosclerosis	Kang J.H. et al., 2006
U937 cells	Human	2-DE/LC-MS/MS	1340	nucleophosmin; serum albumin; serum protein 90K; Hsp70; fumarase; Prdx 1; transgelin 2; aldolase A; GAPDH; hnRNP; calreticulin precursor	Atherosclerosis	Yu Y.L. et al., 2003
Monocyte-derived macrophages	Human	nanoLC-MS/MS	5102	MRC1; APOL2; APOL3, IDO1, GBP1, GBP5, CD274, STAT1, STAT2, WARS, TAP2; NEDD8 ultimate buster 1; CD38; HLA-DRA; HLA-DRB1; HLA-DR3; HLA-DPA1; HLA-DPB1; CD74; ALOX15; CRABP2; CD209; NAIP; CD163; FPR1; coagulation factor XIII; CD32	Atherosclerosis	Court M. et al., 2017
Monocyte-derived macrophages	Human	label-free LC-MS^E^	132	Rab3A; FABP4; TGM-2; HSP70; HSP90; actin, tubulin, myosin	Atherosclerosis	Eligini S. et al., 2015

*Abbreviations:* LC-MS/MS, liquid chromatography coupled with tandem mass spectrometry; 2-DE, includes two-dimensional gel electrophoresis; MALDI-TOF MS, matrix-assisted laser desorption/ionization time-of-flight MS; ELISA, enzyme-linked immunosorbent assay; iTRAQ, isobaric tags for relative and absolute quantitation; ALG-2, apoptosis-linked gene 2; TSP-2, trombospondin-2; MnSOD, manganese superoxide dismutase; apoE, apolipoprotein E; apoB100, apolipoprotein B100; PTP1C, protein-tyrosine phosphatase 1C; GSK-3β, glycogen synthase kinase-3β; Gads, Grb2-like adaptor protein; GIT1, G protein-coupled receptor kinase-interacting protein; HIF-1α, hypoxia-inducible factor-1α; JAM-1, junctional adhesion molecule-1; JNK, c-Jun N-terminal kinase; TNF-α, tumor necrosis factor-α; TOPO-II-α, topoisomerase-II-α; TRAF4, TNF receptor-activating factor-4; S100A12, S100 calcium-binding protein A12; CKB, creatine kinase B; CEMIP, cell-migration-inducing hyaluronan binding protein; LAMC1, laminin subunit gamma 1; LAMA5, laminin subunit alpha-5; LAMB2, laminin subunit beta 2; HSPG2, basement-membrane-specific heparan sulfate proteoglycan core protein; NID1, nidogen 1; AGRN, agrin; NID2, nidogen 2; COL18A1, collagen type XVIII alpha 1 chain; ELN, elastin; FBLN5, fibulin 5; LTBP4, latent-transforming growth factor beta-binding protein 4; MFAP4, microfibril-associated glycoprotein 4; BCAM, basal cell adhesion molecule; LRG, leucin-rich α-2-glycoprotein; apoA-I, apolipoprotein A-I; protein HC, complex-forming glycoprotein heterogeneous in charge HC; SAP, serum amyloid P component; HSP27, heat shock protein 27; ZAG, zinc-alpha-2-glycoprotein; ecSOD, extracellular superoxide dismutase; Prdx2, peroxiredoxin 2; CA1, carbonic anhydrase 1; HSP70, heat shock protein 70; SDF1-α, stromal-cell-derived factor 1α; TGF-β, transforming growth factor beta; PEDF, pigment epithelium-derived factor; MFG-E8, milk fat globule epithelial growth factor-8; MAGP-4, microfibrillar-associated glycoprotein 4; LAR(PTP), leukocyte-antigen-related protein phosphatase; AKAP149, A-kinase anchor protein 149; RanBP1, ran-specific GTPase-activating protein; NM23-H1, nucleoside diphosphate kinase A; SIPA, signal-induced proliferation-associated gene; CDK-1, cyclin-dependent kinase 1; CLIP115, cytoplasmic linker protein 115; TFII-I, transcription factor II-I; COX-2, cyclooxygenase-2; TRADD, tumor necrosis factor receptor type 1-associated DEATH domain protein; MGA6, meningioma-expressed antigen 6; PP2A, serine/threonine protein phosphatase 2A; TFPI2, tissue factor pathway inhibitor 2; HFR, histamine-releasing factor; Prdx1, peroxiredoxin 1; GAPDH, glyceraldehyde-3-phospate dehydrogenase; hnRNP, heterogeneous nuclear ribonucleoprotein; MRC1, macrophage mannose receptor 1; APOL2, apolipoprotein L2; APOL3, apolipoprotein L3; IDO1, indoleamine 2,3-dioxygenase 1; GBP1, guanylate binding protein 1; GBP5, guanylate binding protein 5; CD274, programmed cell death 1 ligand 1; STAT2, signal transducer and activator of transcription 2; WARS, tryptophan-tRNA ligase; TAP2, antigen peptide transporter 2; ALOX15, arachidonate15-lipoxygenase; CRABP2, cellular retinoic acid-binding protein2; NAIP, baculoviral IAP repeat-containing protein 1; FPR1, formyl peptide receptor 1; FABP4, fatty acid-binding protein; TGM-2, transglutaminase-2.

## Data Availability

Not applicable.

## References

[1] Tsao C.W., Aday A.W., Almarzooq Z.I., Alonso A., Beaton A.Z., Bittencourt M.S., Boehme A.K., Buxton A.E., Carson A.P., Commodore-Mensah Y. (2022). Heart Disease and Stroke Statistics-2022 Update: A Report From the American Heart Association. Circulation.

[2] Libby P., Ridker P.M., Maseri A. (2002). Inflammation and atherosclerosis. Circulation.

[3] McLaren J.E., Michael D.R., Ashlin T.G., Ramji D.P. (2011). Cytokines, macrophage lipid metabolism and foam cells: Implications for cardiovascular disease therapy. Prog. Lipid Res..

[4] Bobryshev Y.V. (2006). Monocyte recruitment and foam cell formation in atherosclerosis. Micron.

[5] Bentzon J.F., Otsuka F., Virmani R., Falk E. (2014). Mechanisms of plaque formation and rupture. Circ. Res..

[6] Finn A.V., Nakano M., Narula J., Kolodgie F.D., Virmani R. (2010). Concept of vulnerable/unstable plaque. Arter. Thromb. Vasc. Biol..

[7] Lin P., Ji H.H., Li Y.J., Guo S.D. (2021). Macrophage Plasticity and Atherosclerosis Therapy. Front. Mol. Biosci..

[8] Farahi L., Sinha S.K., Lusis A.J. (2021). Roles of Macrophages in Atherogenesis. Front. Pharm..

[9] Robbins C.S., Hilgendorf I., Weber G.F., Theurl I., Iwamoto Y., Figueiredo J.L., Gorbatov R., Sukhova G.K., Gerhardt L.M., Smyth D. (2013). Local proliferation dominates lesional macrophage accumulation in atherosclerosis. Nat. Med..

[10] Gui T., Shimokado A., Sun Y., Akasaka T., Muragaki Y. (2012). Diverse roles of macrophages in atherosclerosis: From inflammatory biology to biomarker discovery. Mediat. Inflamm..

[11] Barrett T.J. (2020). Macrophages in Atherosclerosis Regression. Arter. Thromb. Vasc. Biol..

[12] Gordon S. (2003). Alternative activation of macrophages. Nat. Rev. Immunol..

[13] Mosser D.M., Edwards J.P. (2008). Exploring the full spectrum of macrophage activation. Nat. Rev. Immunol..

[14] Gordon S., Martinez F.O. (2010). Alternative activation of macrophages: Mechanism and functions. Immunity.

[15] Murray P.J., Wynn T.A. (2011). Protective and pathogenic functions of macrophage subsets. Nat. Rev. Immunol..

[16] Wang L.X., Zhang S.X., Wu H.J., Rong X.L., Guo J. (2019). M2b macrophage polarization and its roles in diseases. J. Leukoc. Biol..

[17] Zizzo G., Hilliard B.A., Monestier M., Cohen P.L. (2012). Efficient clearance of early apoptotic cells by human macrophages requires M2c polarization and MerTK induction. J. Immunol..

[18] Grinberg S., Hasko G., Wu D., Leibovich S.J. (2009). Suppression of PLCbeta2 by endotoxin plays a role in the adenosine A(2A) receptor-mediated switch of macrophages from an inflammatory to an angiogenic phenotype. Am. J. Pathol..

[19] Ferrante C.J., Pinhal-Enfield G., Elson G., Cronstein B.N., Hasko G., Outram S., Leibovich S.J. (2013). The adenosine-dependent angiogenic switch of macrophages to an M2-like phenotype is independent of interleukin-4 receptor alpha (IL-4Ralpha) signaling. Inflammation.

[20] Kadl A., Meher A.K., Sharma P.R., Lee M.Y., Doran A.C., Johnstone S.R., Elliott M.R., Gruber F., Han J., Chen W. (2010). Identification of a novel macrophage phenotype that develops in response to atherogenic phospholipids via Nrf2. Circ. Res..

[21] Eligini S., Brambilla M., Banfi C., Camera M., Sironi L., Barbieri S.S., Auwerx J., Tremoli E., Colli S. (2002). Oxidized phospholipids inhibit cyclooxygenase-2 in human macrophages via nuclear factor-kappaB/IkappaB- and ERK2-dependent mechanisms. Cardiovasc. Res..

[22] Linton M.F., Fazio S. (2004). Cyclooxygenase-2 and inflammation in atherosclerosis. Curr. Opin. Pharm..

[23] Nielsen M.J., Moller H.J., Moestrup S.K. (2010). Hemoglobin and heme scavenger receptors. Antioxid. Redox. Signal..

[24] Boyle J.J. (2012). Heme and haemoglobin direct macrophage Mhem phenotype and counter foam cell formation in areas of intraplaque haemorrhage. Curr. Opin. Lipidol..

[25] Boyle J.J., Johns M., Kampfer T., Nguyen A.T., Game L., Schaer D.J., Mason J.C., Haskard D.O. (2012). Activating transcription factor 1 directs Mhem atheroprotective macrophages through coordinated iron handling and foam cell protection. Circ. Res..

[26] Philippidis P., Mason J.C., Evans B.J., Nadra I., Taylor K.M., Haskard D.O., Landis R.C. (2004). Hemoglobin scavenger receptor CD163 mediates interleukin-10 release and heme oxygenase-1 synthesis: Antiinflammatory monocyte-macrophage responses in vitro, in resolving skin blisters in vivo, and after cardiopulmonary bypass surgery. Circ. Res..

[27] Erbel C., Tyka M., Helmes C.M., Akhavanpoor M., Rupp G., Domschke G., Linden F., Wolf A., Doesch A., Lasitschka F. (2015). CXCL4-induced plaque macrophages can be specifically identified by co-expression of MMP7+S100A8+ in vitro and in vivo. Innate Immun..

[28] Gleissner C.A., Shaked I., Erbel C., Bockler D., Katus H.A., Ley K. (2010). CXCL4 downregulates the atheroprotective hemoglobin receptor CD163 in human macrophages. Circ. Res..

[29] Stoger J.L., Gijbels M.J., van der Velden S., Manca M., van der Loos C.M., Biessen E.A., Daemen M.J., Lutgens E., de Winther M.P. (2012). Distribution of macrophage polarization markers in human atherosclerosis. Atherosclerosis.

[30] Cho K.Y., Miyoshi H., Kuroda S., Yasuda H., Kamiyama K., Nakagawara J., Takigami M., Kondo T., Atsumi T. (2013). The phenotype of infiltrating macrophages influences arteriosclerotic plaque vulnerability in the carotid artery. J. Stroke Cereb. Dis..

[31] Chinetti-Gbaguidi G., Colin S., Staels B. (2015). Macrophage subsets in atherosclerosis. Nat. Rev. Cardiol..

[32] De Gaetano M., Crean D., Barry M., Belton O. (2016). M1- and M2-Type Macrophage Responses Are Predictive of Adverse Outcomes in Human Atherosclerosis. Front. Immunol..

[33] Landis R.C., Philippidis P., Domin J., Boyle J.J., Haskard D.O. (2013). Haptoglobin Genotype-Dependent Anti-Inflammatory Signaling in CD163(+) Macrophages. Int. J. Inflam..

[34] Finn A.V., Nakano M., Polavarapu R., Karmali V., Saeed O., Zhao X., Yazdani S., Otsuka F., Davis T., Habib A. (2012). Hemoglobin directs macrophage differentiation and prevents foam cell formation in human atherosclerotic plaques. J. Am. Coll. Cardiol..

[35] Skuratovskaia D., Vulf M., Khaziakhmatova O., Malashchenko V., Komar A., Shunkin E., Shupletsova V., Goncharov A., Urazova O., Litvinova L. (2020). Tissue-Specific Role of Macrophages in Noninfectious Inflammatory Disorders. Biomedicines.

[36] Eligini S., Brioschi M., Fiorelli S., Tremoli E., Banfi C., Colli S. (2015). Human monocyte-derived macrophages are heterogenous: Proteomic profile of different phenotypes. J. Proteom..

[37] Eligini S., Brioschi M., Fiorelli S., Tremoli E., Colli S., Banfi C. (2015). Data for proteomic analysis of Human monocyte-derived macrophages. Data Brief.

[38] Lee C.W., Hwang I., Park C.S., Lee H., Park D.W., Kang S.J., Lee S.W., Kim Y.H., Park S.W., Park S.J. (2013). Macrophage heterogeneity of culprit coronary plaques in patients with acute myocardial infarction or stable angina. Am. J. Clin. Pathol..

[39] Shankman L.S., Gomez D., Cherepanova O.A., Salmon M., Alencar G.F., Haskins R.M., Swiatlowska P., Newman A.A., Greene E.S., Straub A.C. (2015). KLF4-dependent phenotypic modulation of smooth muscle cells has a key role in atherosclerotic plaque pathogenesis. Nat. Med..

[40] Njoroge J.M., Mitchell L.B., Centola M., Kastner D., Raffeld M., Miller J.L. (2001). Characterization of viable autofluorescent macrophages among cultured peripheral blood mononuclear cells. Cytometry.

[41] Albaghdadi M.S., Ikegami R., Kassab M.B., Gardecki J.A., Kunio M., Chowdhury M.M., Khamis R., Libby P., Tearney G.J., Jaffer F.A. (2021). Near-Infrared Autofluorescence in Atherosclerosis Associates With Ceroid and Is Generated by Oxidized Lipid-Induced Oxidative Stress. Arter. Thromb. Vasc. Biol..

[42] Li W., Ostblom M., Xu L.H., Hellsten A., Leanderson P., Liedberg B., Brunk U.T., Eaton J.W., Yuan X.M. (2006). Cytocidal effects of atheromatous plaque components: The death zone revisited. Faseb J..

[43] Htun N.M., Chen Y.C., Lim B., Schiller T., Maghzal G.J., Huang A.L., Elgass K.D., Rivera J., Schneider H.G., Wood B.R. (2017). Near-infrared autofluorescence induced by intraplaque hemorrhage and heme degradation as marker for high-risk atherosclerotic plaques. Nat. Commun..

[44] Eijgelaar W.J., Horrevoets A.J., Bijnens A.P., Daemen M.J., Verhaegh W.F. (2010). Equivalence testing in microarray analysis: Similarities in the transcriptome of human atherosclerotic and nonatherosclerotic macrophages. Physiol. Genom..

[45] Chai J.T., Ruparelia N., Goel A., Kyriakou T., Biasiolli L., Edgar L., Handa A., Farrall M., Watkins H., Choudhury R.P. (2018). Differential Gene Expression in Macrophages From Human Atherosclerotic Plaques Shows Convergence on Pathways Implicated by Genome-Wide Association Study Risk Variants. Arter. Thromb. Vasc. Biol..

[46] Shen Y., Xu L.R., Tang X., Lin C.P., Yan D., Xue S., Qian R.Z., Guo D.Q. (2021). Identification of potential therapeutic targets for atherosclerosis by analysing the gene signature related to different immune cells and immune regulators in atheromatous plaques. Bmc Med. Genom..

[47] Verma S., Kumar A., Narang R., Bisoi A.K., Mitra D.K. (2022). Signature transcriptome analysis of stage specific atherosclerotic plaques of patients. Bmc Med. Genom..

[48] Chistiakov D.A., Bobryshev Y.V., Orekhov A.N. (2015). Changes in transcriptome of macrophages in atherosclerosis. J. Cell Mol. Med..

[49] Willemsen L., de Winther M.P. (2020). Macrophage subsets in atherosclerosis as defined by single-cell technologies. J. Pathol..

[50] Fernandez D.M., Rahman A.H., Fernandez N.F., Chudnovskiy A., Amir E.D., Amadori L., Khan N.S., Wong C.K., Shamailova R., Hill C.A. (2019). Single-cell immune landscape of human atherosclerotic plaques. Nat. Med..

[51] Eberhardt N., Giannarelli C. (2022). How Single-Cell Technologies Have Provided New Insights Into Atherosclerosis. Arter. Thromb. Vasc. Biol..

[52] Gerner M.Y., Kastenmuller W., Ifrim I., Kabat J., Germain R.N. (2012). Histo-cytometry: A method for highly multiplex quantitative tissue imaging analysis applied to dendritic cell subset microanatomy in lymph nodes. Immunity.

[53] Macklin A., Khan S., Kislinger T. (2020). Recent advances in mass spectrometry based clinical proteomics: Applications to cancer research. Clin. Proteom..

[54] Castagna A., Polati R., Bossi A.M., Girelli D. (2012). Monocyte/macrophage proteomics: Recent findings and biomedical applications. Expert Rev. Proteom..

[55] Banfi C., Baetta R., Gianazza E., Tremoli E. (2017). Technological advances and proteomic applications in drug discovery and target deconvolution: Identification of the pleiotropic effects of statins. Drug Discov. Today.

[56] Matallana-Surget S., Leroy B., Wattiez R. (2010). Shotgun proteomics: Concept, key points and data mining. Expert Rev. Proteom..

[57] Meyer J.G. (2021). Qualitative and Quantitative Shotgun Proteomics Data Analysis from Data-Dependent Acquisition Mass Spectrometry. Methods Mol. Biol..

[58] Gevaert K., Impens F., Ghesquiere B., Van Damme P., Lambrechts A., Vandekerckhove J. (2008). Stable isotopic labeling in proteomics. Proteomics.

[59] Old W.M., Meyer-Arendt K., Aveline-Wolf L., Pierce K.G., Mendoza A., Sevinsky J.R., Resing K.A., Ahn N.G. (2005). Comparison of label-free methods for quantifying human proteins by shotgun proteomics. Mol. Cell Proteom..

[60] Gianazza E., Tremoli E., Banfi C. (2014). The selected reaction monitoring/multiple reaction monitoring-based mass spectrometry approach for the accurate quantitation of proteins: Clinical applications in the cardiovascular diseases. Expert Rev. Proteom..

[61] Tuomisto T.T., Riekkinen M.S., Viita H., Levonen A.L., Yla-Herttuala S. (2005). Analysis of gene and protein expression during monocyte-macrophage differentiation and cholesterol loading--cDNA and protein array study. Atherosclerosis.

[62] De la Cuesta F., Alvarez-Llamas G., Gil-Dones F., Martin-Rojas T., Zubiri I., Pastor C., Barderas M.G., Vivanco F. (2009). Tissue proteomics in atherosclerosis: Elucidating the molecular mechanisms of cardiovascular diseases. Expert Rev. Proteom..

[63] Martinet W., Schrijvers D.M., De Meyer G.R., Herman A.G., Kockx M.M. (2003). Western array analysis of human atherosclerotic plaques: Downregulation of apoptosis-linked gene 2. Cardiovasc. Res..

[64] Slevin M., Elasbali A.B., Miguel Turu M., Krupinski J., Badimon L., Gaffney J. (2006). Identification of differential protein expression associated with development of unstable human carotid plaques. Am. J. Pathol..

[65] Seimon T., Tabas I. (2009). Mechanisms and consequences of macrophage apoptosis in atherosclerosis. J. Lipid Res..

[66] Bagnato C., Thumar J., Mayya V., Hwang S.I., Zebroski H., Claffey K.P., Haudenschild C., Eng J.K., Lundgren D.H., Han D.K. (2007). Proteomics analysis of human coronary atherosclerotic plaque: A feasibility study of direct tissue proteomics by liquid chromatography and tandem mass spectrometry. Mol. Cell. Proteom..

[67] Bao M.H., Zhang R.Q., Huang X.S., Zhou J., Guo Z., Xu B.F., Liu R. (2021). Transcriptomic and Proteomic Profiling of Human Stable and Unstable Carotid Atherosclerotic Plaques. Front. Genet..

[68] Farokhzadian J., Mangolian Shahrbabaki P., Bagheri V. (2019). S100A12-CD36 axis: A novel player in the pathogenesis of atherosclerosis?. Cytokine.

[69] Stakhneva E.M., Meshcheryakova I.A., Demidov E.A., Starostin K.V., Sadovski E.V., Peltek S.E., Voevoda M.I., Chernyavskii A.M., Volkov A.M., Ragino Y.I. (2019). A Proteomic Study of Atherosclerotic Plaques in Men with Coronary Atherosclerosis. Diagnostics.

[70] Haversen L., Sundelin J.P., Mardinoglu A., Rutberg M., Stahlman M., Wilhelmsson U., Hulten L.M., Pekny M., Fogelstrand P., Bentzon J.F. (2018). Vimentin deficiency in macrophages induces increased oxidative stress and vascular inflammation but attenuates atherosclerosis in mice. Sci. Rep..

[71] Vaisar T., Hu J.H., Airhart N., Fox K., Heinecke J., Nicosia R.F., Kohler T., Potter Z.E., Simon G.M., Dix M.M. (2020). Parallel Murine and Human Plaque Proteomics Reveals Pathways of Plaque Rupture. Circ. Res..

[72] Stastna M., Van Eyk J.E. (2012). Secreted proteins as a fundamental source for biomarker discovery. Proteomics.

[73] Brioschi M., Lento S., Tremoli E., Banfi C. (2013). Proteomic analysis of endothelial cell secretome: A means of studying the pleiotropic effects of Hmg-CoA reductase inhibitors. J. Proteom..

[74] Moore K.J., Sheedy F.J., Fisher E.A. (2013). Macrophages in atherosclerosis: A dynamic balance. Nat. Rev. Immunol..

[75] Ait-Oufella H., Taleb S., Mallat Z., Tedgui A. (2011). Recent advances on the role of cytokines in atherosclerosis. Arter. Thromb. Vasc. Biol..

[76] Niu C., Wang X., Zhao M., Cai T., Liu P., Li J., Willard B., Zu L., Zhou E., Li Y. (2016). Macrophage Foam Cell-Derived Extracellular Vesicles Promote Vascular Smooth Muscle Cell Migration and Adhesion. J. Am. Heart Assoc..

[77] Duran M.C., Martin-Ventura J.L., Mohammed S., Barderas M.G., Blanco-Colio L.M., Mas S., Moral V., Ortega L., Tunon J., Jensen O.N. (2007). Atorvastatin modulates the profile of proteins released by human atherosclerotic plaques. Eur. J. Pharm..

[78] Duran M.C., Martin-Ventura J.L., Mas S., Barderas M.G., Darde V.M., Jensen O.N., Egido J., Vivanco F. (2007). Characterization of the human atheroma plaque secretome by proteomic analysis. Methods Mol. Biol..

[79] Li W., Dalen H., Eaton J.W., Yuan X.M. (2001). Apoptotic death of inflammatory cells in human atheroma. Arter. Thromb. Vasc. Biol..

[80] Auguet T., Aragones G., Guiu-Jurado E., Berlanga A., Curriu M., Martinez S., Alibalic A., Aguilar C., Camara M.L., Hernandez E. (2016). Adipo/cytokines in atherosclerotic secretomes: Increased visfatin levels in unstable carotid plaque. Bmc Cardiovasc. Disord..

[81] Dahl T.B., Yndestad A., Skjelland M., Oie E., Dahl A., Michelsen A., Damas J.K., Tunheim S.H., Ueland T., Smith C. (2007). Increased expression of visfatin in macrophages of human unstable carotid and coronary atherosclerosis: Possible role in inflammation and plaque destabilization. Circulation.

[82] Zhou F., Pan Y., Huang Z., Jia Y., Zhao X., Chen Y., Diao J., Wan Q., Cui X. (2013). Visfatin induces cholesterol accumulation in macrophages through up-regulation of scavenger receptor-A and CD36. Cell Stress Chaperones.

[83] Aragones G., Auguet T., Guiu-Jurado E., Berlanga A., Curriu M., Martinez S., Alibalic A., Aguilar C., Hernandez E., Camara M.L. (2016). Proteomic Profile of Unstable Atheroma Plaque: Increased Neutrophil Defensin 1, Clusterin, and Apolipoprotein E Levels in Carotid Secretome. J. Proteome Res..

[84] Quinn K.L., Henriques M., Tabuchi A., Han B., Yang H., Cheng W.E., Tole S., Yu H., Luo A., Charbonney E. (2011). Human neutrophil peptides mediate endothelial-monocyte interaction, foam cell formation, and platelet activation. Arter. Thromb. Vasc. Biol..

[85] Yanni A.E., Agrogiannis G., Gkekas C., Perrea D. (2014). Clusterin/Apolipoprotein J immunolocalization on carotid artery is affected by TNF-alpha, cigarette smoking and anti-platelet treatment. Lipids Health Dis..

[86] Bellosta S., Mahley R.W., Sanan D.A., Murata J., Newland D.L., Taylor J.M., Pitas R.E. (1995). Macrophage-specific expression of human apolipoprotein E reduces atherosclerosis in hypercholesterolemic apolipoprotein E-null mice. J. Clin. Investig..

[87] Li P., Hao Z., Wu J., Ma C., Xu Y., Li J., Lan R., Zhu B., Ren P., Fan D. (2021). Comparative Proteomic Analysis of Polarized Human THP-1 and Mouse RAW264.7 Macrophages. Front. Immunol..

[88] He L., Jhong J.H., Chen Q., Huang K.Y., Strittmatter K., Kreuzer J., DeRan M., Wu X., Lee T.Y., Slavov N. (2021). Global characterization of macrophage polarization mechanisms and identification of M2-type polarization inhibitors. Cell. Rep..

[89] Zhang L., Lun Y., Yan D., Yu L., Ma W., Du B., Zhu X. (2007). Proteomic analysis of macrophages: A new way to identify novel cell-surface antigens. J. Immunol. Methods.

[90] Becker L., Liu N.C., Averill M.M., Yuan W., Pamir N., Peng Y., Irwin A.D., Fu X., Bornfeldt K.E., Heinecke J.W. (2012). Unique proteomic signatures distinguish macrophages and dendritic cells. PLoS ONE.

[91] Yu Y.L., Huang Z.Y., Yang P.Y., Rui Y.C., Yang P.Y. (2003). Proteomic studies of macrophage-derived foam cell from human U937 cell line using two-dimensional gel electrophoresis and tandem mass spectrometry. J. Cardiovasc. Pharm..

[92] Kang J.H., Kim H.T., Choi M.S., Lee W.H., Huh T.L., Park Y.B., Moon B.J., Kwon O.S. (2006). Proteome analysis of human monocytic THP-1 cells primed with oxidized low-density lipoproteins. Proteomics.

[93] Court M., Petre G., Atifi M.E., Millet A. (2017). Proteomic Signature Reveals Modulation of Human Macrophage Polarization and Functions Under Differing Environmental Oxygen Conditions. Mol. Cell Proteom..

[94] Di Rosa M., Malaguarnera G., De Gregorio C., D’Amico F., Mazzarino M.C., Malaguarnera L. (2013). Modulation of chitotriosidase during macrophage differentiation. Cell. Biochem. Biophys..

[95] Li W., Sultana N., Siraj N., Ward L.J., Pawlik M., Levy E., Jovinge S., Bengtsson E., Yuan X.M. (2016). Autophagy dysfunction and regulatory cystatin C in macrophage death of atherosclerosis. J. Cell. Mol. Med..

[96] Magnusson L.U., Lundqvist A., Karlsson M.N., Skalen K., Levin M., Wiklund O., Boren J., Hulten L.M. (2012). Arachidonate 15-lipoxygenase type B knockdown leads to reduced lipid accumulation and inflammation in atherosclerosis. PLoS ONE.

[97] Wigren M., Rattik S., Yao Mattisson I., Tomas L., Gronberg C., Soderberg I., Alm R., Sundius L., Ljungcrantz I., Bjorkbacka H. (2019). Lack of Ability to Present Antigens on Major Histocompatibility Complex Class II Molecules Aggravates Atherosclerosis in ApoE(-/-) Mice. Circulation.

[98] Murugesan G., Davidson L., Jannetti L., Crocker P.R., Weigle B. (2022). Quantitative Proteomics of Polarised Macrophages Derived from Induced Pluripotent Stem Cells. Biomedicines.

[99] Cochain C., Vafadarnejad E., Arampatzi P., Pelisek J., Winkels H., Ley K., Wolf D., Saliba A.E., Zernecke A. (2018). Single-Cell RNA-Seq Reveals the Transcriptional Landscape and Heterogeneity of Aortic Macrophages in Murine Atherosclerosis. Circ. Res..

[100] Lin J.D., Nishi H., Poles J., Niu X., McCauley C., Rahman K., Brown E.J., Yeung S.T., Vozhilla N., Weinstock A. (2019). Single-cell analysis of fate-mapped macrophages reveals heterogeneity, including stem-like properties, during atherosclerosis progression and regression. Jci Insight.

[101] Olsen L.R., Leipold M.D., Pedersen C.B., Maecker H.T. (2019). The anatomy of single cell mass cytometry data. Cytom. A.

[102] Tanner S.D., Baranov V.I., Ornatsky O.I., Bandura D.R., George T.C. (2013). An introduction to mass cytometry: Fundamentals and applications. Cancer Immunol. Immunother..

[103] Winkels H., Ehinger E., Vassallo M., Buscher K., Dinh H.Q., Kobiyama K., Hamers A.A.J., Cochain C., Vafadarnejad E., Saliba A.E. (2018). Atlas of the Immune Cell Repertoire in Mouse Atherosclerosis Defined by Single-Cell RNA-Sequencing and Mass Cytometry. Circ. Res..

[104] Seneviratne A., Cave L., Hyde G., Moestrup S.K., Carling D., Mason J.C., Haskard D.O., Boyle J.J. (2021). Metformin directly suppresses atherosclerosis in normoglycaemic mice via haematopoietic adenosine monophosphate-activated protein kinase. Cardiovasc. Res..

[105] Tokutome M., Matoba T., Nakano Y., Okahara A., Fujiwara M., Koga J.I., Nakano K., Tsutsui H., Egashira K. (2019). Peroxisome proliferator-activated receptor-gamma targeting nanomedicine promotes cardiac healing after acute myocardial infarction by skewing monocyte/macrophage polarization in preclinical animal models. Cardiovasc. Res..

[106] Hara T., Fukuda D., Tanaka K., Higashikuni Y., Hirata Y., Nishimoto S., Yagi S., Yamada H., Soeki T., Wakatsuki T. (2015). Rivaroxaban, a novel oral anticoagulant, attenuates atherosclerotic plaque progression and destabilization in ApoE-deficient mice. Atherosclerosis.

[107] Brenner C., Franz W.M., Kuhlenthal S., Kuschnerus K., Remm F., Gross L., Theiss H.D., Landmesser U., Krankel N. (2015). DPP-4 inhibition ameliorates atherosclerosis by priming monocytes into M2 macrophages. Int. J. Cardiol..

[108] Birrer M.J., Moore K.N., Betella I., Bates R.C. (2019). Antibody-Drug Conjugate-Based Therapeutics: State of the Science. J. Natl. Cancer Inst..

[109] Granfeldt A., Hvas C.L., Graversen J.H., Christensen P.A., Petersen M.D., Anton G., Svendsen P., Solling C., Etzerodt A., Tonnesen E. (2013). Targeting dexamethasone to macrophages in a porcine endotoxemic model. Crit. Care Med..

[110] Adair J.R., Howard P.W., Hartley J.A., Williams D.G., Chester K.A. (2012). Antibody-drug conjugates—A perfect synergy. Expert Opin. Biol. Ther..

[111] Desgeorges T., Caratti G., Mounier R., Tuckermann J., Chazaud B. (2019). Glucocorticoids Shape Macrophage Phenotype for Tissue Repair. Front. Immunol..

[112] Thomsen K.L., Moller H.J., Graversen J.H., Magnusson N.E., Moestrup S.K., Vilstrup H., Gronbaek H. (2016). Anti-CD163-dexamethasone conjugate inhibits the acute phase response to lipopolysaccharide in rats. World J. Hepatol..

[113] Svendsen P., Graversen J.H., Etzerodt A., Hager H., Roge R., Gronbaek H., Christensen E.I., Moller H.J., Vilstrup H., Moestrup S.K. (2017). Antibody-Directed Glucocorticoid Targeting to CD163 in M2-type Macrophages Attenuates Fructose-Induced Liver Inflammatory Changes. Mol. Ther. Methods Clin. Dev..

[114] He H., Yuan Q., Bie J., Wallace R.L., Yannie P.J., Wang J., Lancina M.G., Zolotarskaya O.Y., Korzun W., Yang H. (2018). Development of mannose functionalized dendrimeric nanoparticles for targeted delivery to macrophages: Use of this platform to modulate atherosclerosis. Transl. Res..

[115] Davis S.M., Reichel D., Bae Y., Pennypacker K.R. (2018). Leukemia Inhibitory Factor-Loaded Nanoparticles with Enhanced Cytokine Metabolic Stability and Anti-Inflammatory Activity. Pharm. Res..

[116] Hristodorov D., Mladenov R., von Felbert V., Huhn M., Fischer R., Barth S., Thepen T. (2015). Targeting CD64 mediates elimination of M1 but not M2 macrophages in vitro and in cutaneous inflammation in mice and patient biopsies. MAbs.

[117] Akinrinmade O.A., Chetty S., Daramola A.K., Islam M.U., Thepen T., Barth S. (2017). CD64: An Attractive Immunotherapeutic Target for M1-type Macrophage Mediated Chronic Inflammatory Diseases. Biomedicines.

[118] Baetta R., Banfi C. (2019). Dkk (Dickkopf) Proteins. Arter. Thromb. Vasc. Biol..

[119] Bories G.F.P., Leitinger N. (2017). Macrophage metabolism in atherosclerosis. Febs Lett..

